# Decrease in oxidative phosphorylation yield in presence of butyrate in perfused liver isolated from fed rats

**DOI:** 10.1186/1472-6793-7-8

**Published:** 2007-08-28

**Authors:** Jean-Louis Gallis, Pierre Tissier, Henri Gin, Marie-Christine Beauvieux

**Affiliations:** 1 Centre de Résonance Magnétique des Systèmes Biologiques, UMR 5536 CNRS-UB2, 146 rue Léo Saignat, 33076 F-Bordeaux Cedex France; 2 Service de Nutrition et Diabétologie, Hôpital Haut-Lévêque, Avenue de Magellan, F-33604 Pessac France

## Abstract

**Background:**

Butyrate is the main nutrient for the colonocytes but the effect of the fraction reaching the liver is not totally known. A decrease in tissue ATP content and increase in respiration was previously demonstrated when livers were perfused with short-chain fatty acids (SCFA) such as butyrate, or octanoate.

In fed rats the oxidative phosphorylation yield was determined on the whole isolated liver perfused with butyrate in comparison with acetate and octoanoate (3 mmol/L). The rate of ATP synthesis was determined in the steady state by monitoring the rate of ATP loss after inhibition of (i) cytochrome oxidase (oxidative phosphorylation) with KCN (2.5 mmol/L) and (ii) glyceraldehyde 3-phosphate dehydrogenase (glycolysis) with IAA (0.5 mmol/L). The ATP flux, estimated by ^31^P Nuclear Magnetic Resonance, and the measured liver respiration allowed the ATP/O ratio to be determined.

**Results:**

ATP turnover was significantly lower in the presence of butyrate (0.40 ± 0.10 μmoles/min.g, p = 0.001, n = 7) and octanoate (0.56 ± 0.10 μmoles/min.g, p = 0.01, n = 5) than in control (1.09 ± 0.13 μmoles/min.g, n = 7), whereas perfusion with acetate induced no significant decrease (0.76 ± 0.10 μmoles/min.g, n = 7). Mitochondrial oxygen consumption was unchanged in the presence of acetate (1.92 ± 0.16 *vs *1.86 ± 0.16 for control) and significantly increased in the presence of butyrate (p = 0.02) and octanoate (p = 0.0004) (2.54 ± 0.18 and 3.04 ± 0.15 μmoles/min.g, respectively). The oxidative phosphorylation yield (ATP/O ratio) calculated in the whole liver was significantly lower with butyrate (0.07 ± 0.02, p = 0.0006) and octanoate (0.09 ± 0.02, p = 0.005) than in control (0.30 ± 0.05), whereas there was no significant change with acetate (0.20 ± 0.02).

**Conclusion:**

Butyrate or octanoate decrease rather than increase the rate of ATP synthesis, resulting in a decrease in the apparent ATP/O ratio. Butyrate as a nutrient has the same effect as longer chain FA. An effect on the hepatic metabolism should be taken into account when large quantities of SCFA are directly used or obtained during therapeutic or nutritional strategies.

## Background

We have previously reported that unlike acetate, the short-chain fatty acid (SCFA) butyrate enhances the rate of net ATP consumption in isolated perfused liver of rat [1]. However, the contribution of oxidative phosphorylation remains to be demonstrated. SCFAs are physiologically produced in the colon of mammals as a result of microbial fermentation of resistant starch and other dietary fibers. It has been reported in humans [2] that fermentation of 80 g of mainly soluble fibers can theoretically yield 300 to 800 mmol SCFA and human nutritional recommendations are at least 30 g of fiber/day [3]. Three SCFA (acetate, propionate and n-butyrate) account for 83% of all SCFAs produced and they are distributed in a fairly constant ratio of 60:25:15, butyrate accounting for about 13% (from 40 to 100 mmol) [4].

A part of the absorbed SCFAs plays a role in maintaining the functional integrity of colonocytes. Butyrate is the main substrate for the aerobic energy metabolism and a trophic factor of the colonocytes [5,6]. Provision of butyrate alone has been shown to increase mucosal growth and epithelial proliferation in the intestine [7]. In humans with intestinal bowel inflammation or colic resection, local trophicity can be improved by irrigation with SCFA [8] or butyrate alone [9], suggesting their therapeutic interest in humans. Moreover, the properties of butyrate on cell growth and the cell cycle are not strictly restricted to the colonic cells, as butyrate has been used to restore differentiated hepatocyte-specific functions in a human liver cell line [10].

Besides this trophic effect of SCFAs, another part of them reaches the liver *via *the portal vein and is metabolized. A removal by the liver close to 100% of butyrate has been thus evidenced in Wistar rats adapted to a high fiber diet [11] and butyrate was also taken up by the liver at a high rate after intracecal loads in the rat [12].

Acetate and butyrate are oxidized and propionate is a gluconeogenic substrate. However, even fatty acids can indirectly produce a stimulation of gluconeogenesis in livers perfused with gluconeogenic substrates, such as lactate [13]. The liver poorly uses lactate in fed rats, but oleate and mainly octanoate are known to reduce the threshold of the lactate hepatic use in gluconeogenesis [14]. A high production of acetylCoA from octanoate (which is independent of the carnitine acyltransferase system) has been proposed in isolated hepatocytes to explain the drastic increase in the utilization of lactate, compared to longer FFA [14], whereas acetate was practically ineffective in stimulating lactate utilization. The first step in the metabolic pathways of acetate and butyrate is activation in the acyl-CoA derivatives to be used in the cell. All these ATP-consuming steps (activation, neoglucogenesis) need to be compensated under physiological conditions by an increase in ATP synthesis in order to maintain an energetic steady state in the cell. Moreover, acyl-CoA are subjected to the mitochondrial β-oxidation that produces acetyl-CoA and reduced cofactors (NADH+H^+^, FADH_2_). The entry of acetyl-CoA into the Krebs cycle also generates some reduced cofactors. The latter have to be reoxidized by the mitochondrial respiratory chain, which consumes oxygen [15,16]. This reoxidation induces a proton flux occurring across the mitochondrial inner membrane that leads in part to a coupled ATP synthesis by means of oxidative phosphorylation.

Among medium-length FFA, octanoate is usually used to investigate the effect of FFA on energy metabolism and it has been shown to decrease the mitochondrial ATP/O ratio, thus reflecting the oxidative phosphorylation yield [17]. However, few studies have been devoted to butyrate which, unlike acetate, enhances the rate of net ATP consumption in perfused isolated rat liver [1].

Evaluation of the ATP/O ratio in the whole organ requires the determination of both oxygen consumption and the rate of ATP synthesis. The *in vivo *ATP turnover can be measured by using NMR magnetization-transfer techniques [18,19], although this method is controversial [20]. From studies combining the estimation of ATP turnover by NMR spectroscopy and respiration by polarography in the whole isolated rat liver, it is possible to calculate the ATP/O ratio [21,22].

Our aim was to investigate the effect of butyrate on the oxidative phosphorylation yield in the whole isolated rat liver. For this purpose, the chemical inhibition of the ATP synthesis pathways (glycolysis and oxidative phosphorylation) made it possible to evaluate the contribution of each pathway to the rate of liver ATP synthesis by monitoring the ATP content using ^31^P NMR [21].

## Results

### Effect of substrate on ATP content

**Figure 1:**
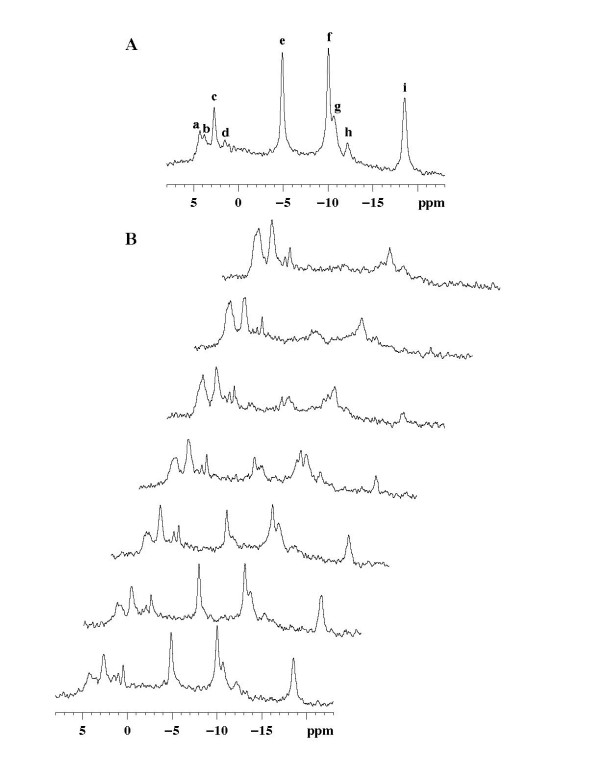
**Typical ^31^P Nuclear Magnetic Resonance spectra of an isolated rat liver**. A: After 30-min control KHB normothermic perfusion. Major resonances are assigned to (a) phosphomonoesters; (b) phosphocholine; (c) intracellular inorganic phosphate (Pi); (d) glycerol-3-phosphoryl ethanolamine and glycerol-3-phosphorylcholine; (e) nucleoside-5'-triphosphates (γNTP) and diphosphates (βNTP); (f) α-NTP and α-NDP; (g) nicotinamide adenine dinucleotide; (h) uridine-5'-diphosphoglucose; (i) βNTP. The reference (methylene-diphosphonic acid) is not shown (18.40 ppm) (number of scans = 148). B: 20 min after a perfusion of butyrate 3 mmol/L, effect (from the bottom to the top) of the simultaneous addition of IAA 0.5 mmol/L (2 min) and KCN 2.5 mmol/L (10 min) (number of scans = 60).

A typical ^31^P NMR spectrum of an isolated rat liver perfused at 37°C with KHB is shown in Figure 1A. The liver ATP content (2.60 ± 0.05 μmol/g liver ww; n = 34) determined from the β-NTP resonance area was quite stable during the initial 30 min period of KHB perfusion in all groups, with a slow ATP degradation (9.01 ± 0.68 nmol/min.g ww; n = 34). This basal decline in ATP level can be prevented by addition of glucose 30 mmol/L with or without insulin [23] leading to suggest its metabolic cause and not a loss of ATP by leakage. Because of the slow ATP degradation, the ATP content was considered near the steady state for short-lasting perfusion (<10 min). This basal ATP consumption led to an ATP level of approximately 72 ± 3% of the initial content after a total of 100 min KHB perfusion. Livers were also perfused with acetate, butyrate or octanoate. The addition of 3 mmol/L of acetate induced a decrease in the ATP content and led to a new steady state of ATP content stabilized at 59 ± 4.6% after 20–30 minutes (p = 0.03 *vs *KHB). The addition of 3 mmol/L of butyrate or octanoate induced a more rapid decrease in ATP content and led to a new steady state of ATP content at a level of 42 ± 4.9% for butyrate and 43 ± 5% for octanoate after 20–30 minutes (both p < 0.001 *vs *KHB). The observed decrease in the ATP content was not reversible after removal of the substrates [1].

This result confirmed a different effect on ATP content of two groups of fatty acids [1]: (i) acetate, which is not concerned by the β-oxidation pathway, did not significantly affect the net ATP consumption whereas the ATP content was significantly decreased after 20–30 min perfusion, and (ii) butyrate and octanoate, which are oxidized in the β-oxidation pathway, induced a large decrease in ATP content due to a significant increase in the net ATP consumption.

### Effect of substrate on ATP turnover: calculation of ATP synthesis rates

#### KHB group (Figure 2A)

**Figure 2:**
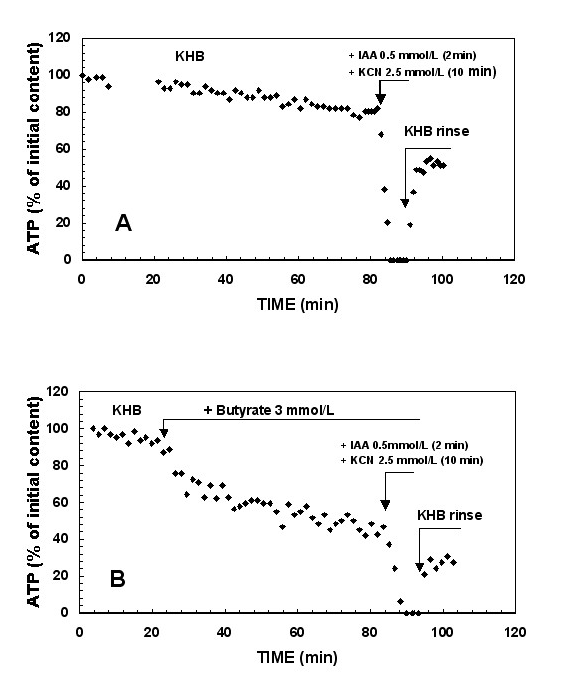
**Time course of liver ATP content throughout the entire protocol perfusion**. Simultaneous inhibition of glycolytic ATP synthesis by IAA (0.5 mmol/L, 2 min) and of ATP mitochondrial synthesis by KCN (2.5 mmol/L, 10 min) for two typical experiments performed in presence of (A) Krebs-Henseleit Buffer and (B) butyrate (3 mmol/L). The results are expressed as percent of ATP content, the 100% value being at the beginning of KHB perfusion (2.60 ± 0.05 μmol/g liver wet weight).

After the ATP steady state was reached, the inhibition of both oxidative phosphorylation and glycolysis (IAA + KCN addition) induced a drastic fall in ATP content reaching zero within 2–3 min at a rate of 1.09 ± 0.13 μmole/min.g (n = 7) (Table 1) in the whole organ. This rate was 100-fold faster than the rate of basal ATP consumption (considered as negligible).

**Table 1:** Calculation of mitochondrial ATP synthesis yield in isolated liver perfused with Krebs-Henseleit Buffer or substrates

	**ATP synthesis rate**	**VO**_2_	**ATP/O**
**KHB**	1.09 ± 0.13	1.86 ± 0.16	0.30 ± 0.05
(n = 7)	-	-	-
**Acetate**	0.76 ± 0.10	1.92 ± 0.16	0.20 ± 0.02
(n = 7)	NS	NS	NS
**Butyrate**	0.40 ± 0.10*	2.54 ± 0.18*	0.07 ± 0.02**
(n = 7)	p = 0.001	p = 0.02	p = 0.0006
**Octanoate**	0.56 ± 0.10***	3.04 ± 0.15***	0.09 ± 0.018***
(n = 5)	p = 0.01	p = 0.0004	p = 0.005

ATP synthesis rate and mitochondrial VO_2 _are expressed in μmole/min.g liver ww. ATP/O = ATP (μmol ATP)/2 VO_2 _(μatoms oxygen). The control group was perfused with Krebs-Henseleit buffer (KHB); the fatty acids were perfused at 3 mmol/L. Values are given as mean ± SEM (Significant ANOVA and t-test).

* p < 0.03 *versus *acetate, ** p = 0.0006 *versus *acetate, *** NS *versus *butyrate.

#### Fatty acid group (Figures 1B and 2B)

When the steady state of ATP content was reached in the presence of each fatty acid (20–30 min perfusion), the total ATP synthesis rate was calculated when IAA + KCN were added. The values gave an ATP synthesis rates of 0.76 ± 0.10, 0.40 ± 0.10 and 0.56 ± 0.10 μmole/min.g for acetate (n = 7), butyrate (n = 7) and octanoate (n = 5), respectively. Therefore, there was a significantly lower ATP turnover in the presence of butyrate (p = 0.001) and octanoate (p = 0.01) compared to the KHB group (Table 1).

### O_2 _consumption (VO_2_)

During KHB perfusion (basal condition), mitochondrial liver consumption was 1.86 ± 0.16 μmol oxygen/min.g ww (n = 7). The addition of 3 mmol/L of each substrate (acetate, butyrate and octanoate) induced an increase in oxygen consumption and reached a new steady state for mitochondrial VO_2 _after 15 min of perfusion (Table 1) increasing with chain length, thus confirming previous results [1]. Addition of IAA + KCN induced the total and immediat inhibition of mitochondrial respiration, leading to a decrease in a few seconds in effluent oxygen to a concentration around 24% (0.59 μmol O_2_/min.g ww) of the initial concentration, thus reflecting KCN-insensitive oxygen consumption in agreement with recent results [24]. Moreover, it has been previously demonstrated that only ≈30% of the mitochondrial respiration was devoted to ATP synthesis [24,25].

### Validation of the method of inhibition of ATP synthesis (Table 2)

**Table 2:** Effects of various sequences of ATP synthesis inhibition in KHB group

**Sequences**	**IAA following by KCN**		**KCN following by IAA**		**Simultaneous IAA+KCN**
	***IAA***	***KCN***	***KCN***	***IAA***	***IAA+KCN***

**Residual ATP content (% of the initial value)**	90%	0	20–40%	0	0
**Residual mitochondrial VO**_2_**(% of the initial value)**	110% (transient)	0	0	0	0
Initial rate of ATP hydrolysis*	ND	1.5	1.0	ND	1.0

**Total inhibition of:**	**Glycolysis only**	**OP only**	**OP only**	**Glycolysis only**	**Glycolysis and OP simultaneously**

* arbitrary units; 1.0 corresponding to the rate of ATP decrease in simultaneous IAA+KCN sequence (1.09 ± 0.13 μmol/min.g liver wet weight). OP = oxidative phosphorylation.

When KCN was used alone, a rapid and partial decrease in ATP signal was observed: the ATP content was not totally decreased and remained at a stable level around 20 to 40% of the initial value. However, the total inhibition of mitochondrial respiration in a few seconds indicated that the ATP plateau, observed after addition of KCN alone, probably originated from glycolysis. A subsequent addition of IAA slowly induced total removal of this residual ATP content, confirming this hypothesis.

The comparison of the ATP decline between the inhibition conditions with KCN alone or simultaneous KCN+IAA, showed that the rate of ATP decrease was not changed by the presence of IAA at the onset of inhibition. At first sight, this should mean only a slight contribution of glycolysis to ATP synthesis in the liver.

On the other hand, when glycolysis was previously inhibited by IAA (0.5 mmol/L; 2 min), we observed a slight decrease in ATP content to a new steady state reached within 10 min. This decrease accounted for a maximum of 10% of the initial ATP content, indicating a slight contribution of glycolysis to total ATP synthesis. Moreover, there was a transient (5 min) VO_2 _increase (< 10 ± 2%) which returned to baseline. The further addition of KCN in a delay of 15 min after the addition of IAA induced an immediate and drastic fall in ATP content (to zero) and VO_2_, thereby showing that mitochondrial respiration and ATP synthesis were totally inhibited. In this sequential inhibition, the apparent rate of ATP decrease was at least 1.5-fold faster than that observed during simultaneous KCN+IAA inhibition, indicating a compensatory stimulation of oxidative phosphorylation after the inhibition of glycolysis alone. Therefore the sequential inhibition was not suitable for the purpose of our study.

Finally, in order to avoid overestimation of ATP synthesis flux, the use of this method of inhibition to determine ATP synthesis implies no increase in the activity of mitochondrial ATP-consuming systems consecutively to the inhibitor addition. In the isolated mitochondria, it has been demonstrated that a rapid reduction of the proton motive force following CN^- ^addition induced the change of F_0_–F_1 _ATP synthase activity to ATPase activity, hydrolyzing ATP originated from cytosol *via *the ATP/ADP translocase [26,27]. Hence, we performed complementary kinetic studies of change of whole liver ATP content in presence or in absence of carboxyatractyloside (200 μmol/L), the specific, non competitive and irreversible inhibitor of translocase [28], when KCN and IAA were used. There was no significant difference of the rate of ATP decrease, demonstrating that in response to the large KCN-induced decrease of proton motive force, liver cytosolic ATP was not hydrolysed *in situ *by mitochondria.

### Yield of ATP synthesis: ATP/O ratio (Table 1)

The ATP/O ratios (μmol ATP/μatom oxygen) were calculated from the previous values of ATP synthesis rate and mitochondrial oxygen consumption. Perfusion with butyrate led to an ATP/O ratio significantly lower (p = 0.0006) than that of the control group and similar to those obtained in the presence of octanoate. No significant variation was observed in the presence of acetate compared to control.

These results demonstrated that fatty acids entering the β-oxidation pathway decreased the oxidative phosphorylation yield by 77% and 70% for butyrate and octanoate, respectively, whereas acetate, which enters the TCA cycle directly, had no significant effect.

## Discussion

Butyrate is one of the SCFAs (acetate, butyrate and propionate) produced by the intestinal bacterial fermentation of unabsorbed soluble carbohydrate. After their uptake by the colon, they are metabolized by the colonocytes with an action on cell growth and cell cycle, while the remaining fraction reaches the liver *via *the portal vein where it is metabolized. Moreover, SCFAs (butyrate) are used to supplement total parenteral nutrition in patients with short bowel syndrome or intestinal malabsorption syndromes. Besides their trophic effects on hepatocytes [10], it is of interest to know the influence of these substrates on oxidative phosphorylation since this may reflect the supply of ATP required for the anabolic pathways. To be free of the known reciprocal influences on SCFA on their absorption and on their liver metabolism [29,30], our study concerned only the inherent effect of separate substrates.

Among medium-length FFA, octanoate is usually used to investigate the effect of FFA on energy metabolism and it has been shown to decrease the mitochondrial ATP/O ratio, thus reflecting the oxidative phosphorylation yield [17]. However, few studies have been devoted to butyrate which, unlike acetate, enhances dramatically the rate of net ATP consumption in perfused isolated rat liver [1].

The present work mainly sought to estimate the effects of butyrate on the yield of *in situ *mitochondrial oxidative phosphorylation in perfused rat liver. NRM spectroscopy made it possible to monitor continuously the ATP content and its turnover in real time in a whole organ or organism. The *in vivo *ATP turnover can be measured by using NMR magnetization-transfer techniques [18,19], although this method is controversial [20] leading to a very large overestimation of ATP fluxes in the liver [18]. Indeed, its basis is the use of exchange reactions between terminal phosphate of ATP and inorganic phosphate (Pi) at the level of (i) mitochondrial ATP synthase or (ii) glycolysis (glyceraldehyde-3-phosphate dehydrogenase/phospho-glycerate kinase couple); the phosphate exchange can be increased during the NMR magnetization-transfer experiment without any net ATP synthesis. Nevertheless, from studies combining the estimation of ATP turnover by NMR spectroscopy and respiration by polarography in the whole isolated rat liver, it is possible to calculate the ATP/O ratio [21,22]. The kinetic method used here to evaluate ATP turnover was based on the complete and rapid inhibition of the processes involved in cellular ATP synthesis, namely, oxidative phosphorylation (inhibited by KCN) and glycolysis (inhibited by IAA). The rates of ATP synthesis and consumption were equal at the steady state. During blockage of the ATP synthesis pathways, the ATP concentration in the organ diminished on the basis of the cellular ATP consumption rate. In fact, the transient increase in oxygen uptake upon previous addition of IAA during sequential inhibition (as shown in the complementary experiment) suggests that there is some degree of adjustment to the impaired metabolic state, in the presence or absence of SCFA. Indeed, the apparent ATP synthesis flux was consistently higher when oxidative phosphorylation was inhibited after glycolysis than when their inhibitors were added together or when KCN was used alone (more than 50% increase). This result suggests that when glycolysis was inhibited before oxidative phosphorylation, the mitochondria compensated for the loss of glycolytic ATP. Therefore, the sequential inhibition protocol is not suitable for our purpose and both oxidative phosphorylation and glycolysis must be simultaneously inhibited in order to avoid compensatory and reciprocal stimulation. Even if the IAA inhibition was not immediate, the contribution of glycolysis to ATP content and ATP flux remained less than 10% at the onset of KCN inhibition.

The respiration rate in coupled mitochondria is a function of several factors including the ADP supply, the proton leak rate, the prevailing membrane potential, and the redox potential span between the electron donors and acceptors. In addition, the activity of the respiratory complexes affects the respiration rate. Nevertheless, the contribution of the various components to the flux control of mitochondrial respiration has been a matter of endless debate for several decades. The production of acyl-CoA *via *short-chain acyl-CoA synthase consumes one ATP/mole FFA whatever the chain length. Butyryl-CoA and octanoyl-CoA are then β-oxidized providing acetyl-CoA and reduced cofactors. Acetyl-CoA finally enters the citric cycle. Among various factors, the rate of respiration depends in part on the amount of reduced cofactors (FADH_2 _and NADH+H^+^) produced during β-oxidation and the citric cycle.

The present work confirms that the respiration rate increased with chain length at the concentration used, according to the process of the β-oxidation pathway [31,1], the lowest respiration rate being observed in the presence of acetate. Because respiration was greatly stimulated in the presence of butyrate and octanoate, an increase in coupled ATP synthesis was expected. However, the ATP content (resulting theoretically from both consumption and synthesis) was unexpectedly decreased at the same rate when butyrate and octanoate were added, reaching a plateau after at least 20 minutes, as previously demonstrated [1]. In this condition, glycolysis was probably inhibited since these fatty acids have been shown to inhibit this pathway in isolated hepatocytes [32]. Moreover, fatty acids have been identified as potent activators of hepatic gluconeogenesis in presence of neoglucogenic substrates such as lactate and pyruvate [13,14]. Therefore, we attempted to estimate *in situ *the flux of ATP synthesis originating from mitochondria, counterbalancing all ATP-consuming processes.

The ratio of synthetic flux of ATP to the mitochondrial liver respiration allowed the phosphorylative oxidation yield to be calculated. Butyrate or octanoate reduced the oxidative phosphorylation rate and increased the respiration rate, leading to the decrease in mitochondrial ATP/O ratio. Octonoate and longer-chain FA have been demonstrated to impair mitochondrial activity due to (i) an intrinsic uncoupling of the respiratory chain (direct effect), hence reducing ATP synthesis [17,33] and/or (ii) a change in the proportion of electron supply to the coupling site due to the increase in FADH_2_/NADH+H^+ ^ratio resulting from β-oxidation [17]. Since the respiration rate depends partly and directly on the amount of reduced cofactors, the latter process could explain in part the dependence from the chain length of the decrease in ATP/O ratio. The low values of hepatic ATP/O ratio calculated in the control fed rats demonstrated that the *in situ *mitochondrial activity in the whole liver was slight and phenomenologically near to state IV, as also observed in the mitochondria freshly isolated from the liver of fed rats [34]. These low ATP/O ratios were far from the P/O ratio of 2.48 indirectly estimated in the perfused liver isolated from 24-hr fasted rat [35] and far from the maximal phosphorylation capacity. In our study the addition of butyrate as well as octanoate induced a large decrease in the yield of phosphorylation, implying that substrate oxidation leads to a large enthalpy supply.

## Conclusion

The difference in the hepatic oxidation pathway of the physiological substrates acetate and butyrate could explain their different action on energy metabolism. Butyrate is the main nutrient for the colonocytes but the large remaining fraction reaching the liver [11] has the same effect as longer-chain FFA. An effect on hepatic metabolism has to be taken into account when large quantities of SCFA are found in therapeutic purposes, especially in long-term acarbose treatment of patients with metabolic syndromes such as type 2 diabetes [36] or NASH [37], thereby reducing the risk of cardiovascular events [38]. Acarbose, an alpha-glucosidase inhibitor, reduces postprandial plasma glucose excursions by delaying the absorption of carbohydrate from the small intestine. It increases (i) the amount of starch entering the colon and (ii) butyrate production, as shown in fermented samples collected in humans undergoing this treatment [39,40]. Moreover, long-chain FFA have been shown to play a large part in the pathogenesis of insulin resistance in human obesity [41], in skeletal muscle and liver [42]. The specific question of the role of butyrate in the metabolic syndrome remains to be elucidated.

## Methods

### Chemicals

High-grade chemicals were purchased from Sigma Chemical (St. Louis, Missouri, USA) except where otherwise specified.

### Animals and liver perfusion

Media were diluted daily from concentrated stock solutions. Standard Krebs-Heinseleit buffer (KHB) was composed of (in mmol/L) 120 NaCl, 4.7 KCl, 1.2 MgSO_4_, 25 NaHCO_3_, 1.2 KH_2_PO_4_-K_2_HPO_4_, 1.3 CaCl_2_, 0.3 Na-pyruvate and 2.1 Na-lactate (pH = 7.35 at 37°C). Acetate, butyrate or octanoate were added to the KHB (3 mmol/L for each substrate). This concentration was chosen as we have previously demonstrated that it leads to maximal oxygen consumption and allows the ATP content steady state to be reached more quickly [1].

Male Wistar rats (Centre d'élevage Depré, St Doulchard, France) weighing 80–120 g, were fed *ad libitum *in order to be free of the metabolic effects of the endogenous FA due to lipolysis observed in fasting conditions. The diet contained 76.55% cereals and the daily caloric supply was (kcal/kg) 4.05% lipids, 3.95% fiber, 18.70% protein, 5.35% minerals and 55.85% carbohydrates [1]. Lipid supply consisted of a mixture of saturated and unsaturated long-chain fatty acids (C16 and C18). Rats were anesthetized by intraperitoneal injection of pentobarbital sodium (50 mg/kg of rat), and the technique of liver antegrade perfusion through the portal vein has been described previously [43]. Briefly, the liver (4–6 g) was perfused in normothermic and well oxygenated conditions with KHB at 37°C regulated by a thermostatic bath. The perfused liver was then excised from the rat abdomen and transferred to a 20 mm-diameter NMR cell. All experiments were carried out after a 20–30 min metabolic equilibration period in KHB.

The perfusate was pumped through a Silastic^© ^home-made oxygenator, gassed with 95% O_2 _and 5% CO_2 _(1 bar pressure). Perfusion flow was kept constant (5 ml/min.g liver wet weight) and was sufficient to ensure good oxygenation of the liver. Perfusate temperature and pH were monitored both before entering and after leaving the liver by continuous-flow pH electrodes and temperature electrodes.

### Determination of ATP/O ratio

In order to suppress cytosolic and ATP mitochondrial production, perfusion was performed using both IAA (0.5 mmol/L) to inhibit glyceraldehyde 3-phosphate dehydrogenase (glycolysis) and KCN (2.5 mmol/L) to inhibit cytochrome oxidase (oxidative phosphorylation). In complementary experiments we showed that the addition of 0.5 mmol/L IAA for two minutes is sufficient to prevent glycolysis totally and irreversibly, without preventing the ATP mitochondrial metabolism (data not shown).

Three groups of animals were used:

(i) NMR measurement group: A first group was submitted to continuous NMR measurements in order to study the kinetic of the ATP content: the sequence was as follows: (i) initial KHB metabolic equilibration period (20–30 min), (ii) addition (or not = control) of substrate (3 mmol/L, 40–60 min) and (iii) in presence (or not = control) of substrate, addition of both IAA (0.5 mmol/L, 2 min) and KCN (2.5 mmol/L, 10 min), followed by (iv) a KHB rinse phase.

(ii) Respiratory measurement group: A second group was devoted to respiratory measurements through a similar sequence.

(iii) Both NMR and respiratory measurement group: A third group corresponded to experiments with sequential NMR and respiratory measurements (requiring removal of NMR cell from magnet).

### Validation of the method of ATP synthesis inhibition

Complementary experiments were performed in order to evaluate the effect of the inhibition of the glycolytic pathway on mitochondrial ATP synthesis. For this purpose, three groups of livers were used:

First group: only KCN (2.5 mmol/L, 40 min) was added to inhibit oxidative phosphorylation.

Second group: a sequential inhibition was performed with KCN added firstly (2.5 mmol/L, 40 min) and followed by a delayed (15 min after the onset of KCN addition) IAA addition (0.5 mmol/L, 2 min).

Third group: a sequential inhibition was performed with IAA (0.5 mmol/L, 2 min) added firstly and followed by a delayed (15 min) KCN addition (2.5 mmol/L).

The study complied with the Guide for the Care and Use of Laboratory Animals, U.S. Department of Health and Human Services, N.I.H. Publication No. 85-23, revised (1985).

### ^31^P NMR measurements

NMR measurements on isolated liver were performed on a Bruker DPX-400 wide-bore spectrometer operating at 9.4 T. ^31^P and ^13^C NMR spectra were recorded at 161.9 and 100.6 MHz respectively, with a ^31^P/^13^C double-tuned 20 mm probe. The magnetic field was adjusted to the water proton signal. ^31^P-NMR spectra were obtained without proton decoupling every 1 min and 30 seconds (148 free induction decays, FIDs) throughout the sequence protocol, except during inhibitor addition times, every 20 seconds (40 FIDs) in order to increase the determination accuracy. Radiofrequency pulses of 25 μs duration (70° flip angle) with 0.2 s acquisition time, 0.4 s total recycling time, 10000 Hz spectral width and 4 K data points were used for data acquisition. A Lorentzian line broadening of 15 Hz was applied prior to Fourier transformation for ^31^P NMR spectra. An external reference of 13 μmoles methylene diphosphonic acid (MDPA set at 18.40 ppm) was used to quantify the phosphorylated metabolites.

Hepatic ATP levels were estimated from peak areas and expressed as a percentage of the initial value obtained at the beginning of the 30-min equilibration period in KHB.

### Evaluation of ATP turnover

When the steady state of the ATP content was reached after the addition of substrate, the addition of "IAA+KCN" allowed the ATP turnover to be determined. At the onset of inhibition, the ATP synthesis velocity corresponds to the initial ATP degradation velocity according to: Vs_(t = 0) _= d [ATP]_(t = 0)_/dt with ATP = A.exp(-kt), where k is the constant time and A the liver content at the onset of the addition of IAA+KCN. Vs was expressed in μmoles ATP/min.g wet weight (ww).

### Oxygen consumption measurements

Isolated perfused livers were placed in an NMR cell similar to that used for the NMR experiments. The total oxygen consumption of liver was continuously monitored using O_2 _Clark electrodes, placed in the perfusion circuit just before and after the liver, using an oxygenmeter model 781 (Strathkelvin Instrument, Glasgow, Scotland). Oxygen consumption (μmol O_2_/min.g liver ww) VO_2 _= (perfusate O_2 _content - effluent O_2 _content) × liver flow rate (ml/mn.g ww) was calculated, where perfusate O_2 _content was expressed in μmol/ml. The mitochondrial respiration component of tissue respiration was calculated by subtracting the KCN-insensitive oxygen consumption from the total O_2 _consumption.

### Statistics

All results were expressed as means ± SEM. Statistical analysis was performed with Microsoft^® ^Excel 2000 (Microsoft Corporation). One-way analysis of variance (ANOVA) was performed for all data analyses. A t test was performed following the analysis of variance (p value lower than 0.05 was considered to be significant).

## Abbreviations

ATP- Adenosine triphosphate.

ATP/O- Oxidative phosphorylation yield.

FFA- Free fatty acids.

IAA- Iodacetic acid.

KHB- Krebs Henseleit buffer.

NMR- Nuclear magnetic resonance.

OP- Oxidative phosphorylation.

SCFA- Short-chain free fatty acids.

## Authors' contributions

MCB participated in the design and coordination of the study and was involved in drafting the manuscript.

PT carried out all the experimental procedures.

HG was highly involved in revising the manuscript.

JLG designed the study, carried out the analysis and data interpretation and was involved in revising the manuscript.

All authors read and approved the final manuscript.
